# Relationship Between Maternal Serum Amyloid A Levels and Neonatal Outcomes in Preterm Births

**DOI:** 10.7759/cureus.75452

**Published:** 2024-12-10

**Authors:** Evelina I Chiriac, Simona Cerbu, Florin I Gorun, Liana-Camelia Buhas, Razvan Oros, Buhas A Bogdan, Narcis Vilceanu, Zoran L Popa, Cosmin Citu, Andrei Csep

**Affiliations:** 1 Doctoral School, Faculty of Medicine and Pharmacy, University of Oradea, Oradea, ROU; 2 Department of Radiology and Medical Imaging, “Victor Babes” University of Medicine and Pharmacy, Timisoara, ROU; 3 Department of Obstetrics and Gynecology, Timisoara Clinical Municipal Emergency Hospital, Timisoara, ROU; 4 Department of Morphological Disciplines, Faculty of Medicine and Pharmacy, University of Oradea, Oradea, ROU; 5 Department of Psycho-Neuroscience and Recovery, Faculty of Medicine and Pharmacy, University of Oradea, Oradea, ROU; 6 Department of Obstetrics-Gynecology and Neonatology, “Victor Babes” University of Medicine and Pharmacy, Timisoara, ROU

**Keywords:** maternal inflammation, maternal serum amyloid a, neonatal outcomes, neonatal respiratory distress syndrome, preterm birth

## Abstract

This study investigated the relationship between maternal serum amyloid A (SAA) levels, a biomarker of systemic inflammation, and specific neonatal outcomes in preterm birth (PTB). The study included 66 consecutive pregnant women hospitalized for spontaneous preterm delivery (ranging from 28 to 36 gestational weeks), at the Timisoara Municipal Hospital. The study measured mSAA levels to assess their potential as predictors of fetal outcomes (respiratory distress syndrome [RDS]), as well as their association with APGAR score, neonatal leukocyte count, and C-reactive protein (CRP) levels as indicators of neonatal status and response. SAA levels were measured as part of the study protocol for all participants admitted with spontaneous preterm labor symptoms.

The results showed a significant negative correlation between mSAA and APGAR score (*r* = -0.272, two-tailed *P*-value = 0.027). This finding suggests that elevated maternal inflammation may adversely impact the neonate's condition at birth. Additionally, a moderate positive correlation was observed between SAA and neonatal leukocyte count (*r* = 0.538, *P* < 0.001), reflecting a neonatal inflammatory response. However, SAA was not a significant predictor for RDS (odds ratio [OR] = 1.005, *P* = 0.31). While these findings are promising, they must be interpreted with caution due to limitations such as the small sample size and the cross-sectional nature of the study design.

These results suggest that elevated mSAA levels may be associated with immediate adverse neonatal outcomes, supporting the use of inflammatory biomarkers to identify neonatal risks, but further studies are needed for validation.

## Introduction

Serum amyloid A (SAA) is an acute-phase protein synthesized by the liver in response to inflammation and infection and is considered a valuable marker for systemic inflammation in various pathological contexts, including pregnancy [1]. In the context of pregnancy, SAA levels are predictive of preterm labor [2,3]. Elevated SAA levels are also connected to clinical chorioamnionitis in women with preterm premature rupture of membranes (PPROM) and may serve as a marker for infection and inflammation in high-risk pregnancies [4]. Furthermore, higher SAA levels have been seen in preeclampsia, which correlate with raised levels of C-reactive protein (CRP) [5]. 

Approximately 15 million infants are born preterm each year, accounting for 11.1% of live births worldwide. Preterm birth (PTB) is the primary cause of newborn mortality, accounting for one million deaths a year. It accounts for 18% of the deaths among children under five. Furthermore, PTB is associated with several severe complications, such as respiratory distress syndrome (RDS), neonatal infections, and early metabolic dysfunction [6-9].

Understanding the role of maternal inflammatory biomarkers is a key research direction in elucidating the pathogenic mechanisms involved in PTB and associated complications [10]. 

Maternal inflammation is an important risk factor for several adverse outcomes in newborns, particularly in cases of PTB. It can result from intrauterine infections or inflammatory conditions such as chorioamnionitis, and the consequences for the newborn can include respiratory, neurological, and other health complications [11,12]. However, studies show that intrauterine inflammation (histological chorioamnionitis or funisitis) may reduce the risk of RDS by accelerating fetal lung maturation via enhanced surfactant synthesis. Pro-inflammatory cytokines in intrauterine inflammation (e.g., interleukin-6, prostaglandin E2) stimulate surfactant production, improving lung function [13-15].

The objective of this study is to assess the relationship between maternal SAA (mSAA) levels and neonatal outcomes in PTBs, specifically focusing on RDS. In addition, we aimed to determine the correlation of mSAA with secondary indicators, including APGAR score (AS), newborn leukocyte count, and CRP levels, to improve our understanding of the inflammatory mechanisms precipitating PTB and assess their subsequent effects on immediate neonatal well-being.

Inflammatory pathways are central to the pathogenesis of PTB, mediated by infection, sterile inflammation, and immune dysregulation. Key pathways include nuclear factor kappa-light-chain-enhancer of activated B cells (NF-κB) signaling, cytokine release, and activation of matrix metalloproteinases (MMPs). Cytokines such as interleukin-6 (IL-6) and tumor necrosis factor-alpha (TNF-α) stimulate uterine contractions and cervical ripening. Elevated amniotic IL-6 levels indicate intra-amniotic infection and PTB [16]. Noninfectious stressors, such as oxidative stress and tissue damage, cause the production of danger-associated molecular patterns (DAMPs), which activate inflammatory pathways comparable to those initiated by infection [17]. NF-κB has a major role in both viral and sterile inflammation. It controls the production of cytokines, chemokines, and prostaglandins that facilitate labor [18]. Intra-amniotic inflammation can stimulate the fetal immune system, resulting in increased cytokines such as IL-6 in cord blood. Fetal Inflammatory Response Syndrome (FIRS) raises the likelihood of neurodevelopmental problems and other consequences [19].

## Materials and methods

Study design

This study was a prospective cohort study, conducted to explore the relationship between mSAA levels and various neonatal outcomes, including AS, neonatal leukocyte count, CRP levels, and the occurrence of neonatal RDS. The study included 66 consecutive participants, all women, who were admitted to the Obstetrics Department of Timisoara Municipal Clinical Emergency Hospital between April 1, 2023, and September 30, 2023, with spontaneous preterm labor for the management of their condition. Their newborns were assessed after delivery for relevant outcomes.

The study received ethical approval from the Ethics Committee of Timisoara Municipal Clinical Emergency Hospital (reference number E-1828, dated March 31, 2023). 

Participants

The study included 66 consecutive women who presented to the Obstetrics Department of the Timisoara Municipal Clinical Emergency Hospital with spontaneous preterm labor and subsequently delivered prematurely. The range of gestational ages among participants spanned from 28 to 36 weeks and six days.

Inclusion criteria included the following: (1) pregnant women aged between 18 and 45 years; (2) singleton pregnancy; (3) no known major chronic diseases (hypertensive disorders, diabetes, autoimmune diseases); (4) premature spontaneous birth (defined as labor occurring before 37 weeks' gestation without medical induction).

Exclusion criteria included: (1) acute infections during pregnancy; (2) uncontrolled chronic pathologies (hypertensive disorders, diabetes, autoimmune diseases); (3) multiple pregnancies; (4) iatrogenic PTB due to maternal and fetal complications; and (5) chorioamnionitis.

Data collection

Data were collected from the hospital clinical records, including demographics, medical and obstetric history, and laboratory test results for SAA on admission. 

Neonatal data, including the AS at 1 minute, leukocyte count, CRP levels, and the presence of neonatal RDS, were extracted from clinical records.

This study prospectively measured SAA levels in consecutive women identified with spontaneous preterm labor. Subsequently, only results from women who gave birth prematurely were included in the study.

Variables

PTB was defined as delivery before 37 weeks of gestation.

The primary exposure variable analyzed was the level of mSAA. mSAA levels were quantified on admission using the nephelometry technique. The measurements were taken with the Atellica® NEPH 630 System (Siemens Healthineers, Erlangen, Germany).

Several outcome variables were assessed to understand the impact of SAA levels on neonatal health, including the newborn AS measured at 1 minute after birth, neonatal leukocyte count, neonatal CRP levels, and the presence of neonatal RDS. 

CRP levels were assessed in all preterm infants according to hospital protocols to detect and manage any early signs of infection or inflammation. Blood was collected from patients immediately after birth, before administration of any medication. The serum was separated by centrifugation (2500 rpm for 20 minutes) - CRP test (Enzyme-Linked Immunosorbent Assay [ELISA]) was performed according to standard procedures. 

The leukocyte count was obtained from the complete blood count (CBC), which is routinely performed on all newborns as per hospital protocol.

Bias

To avoid selection bias and enhance the representativeness of our sample, participants were consecutively recruited from the obstetrics department of the Timisoara Municipal Hospital. To mitigate potential measurement biases, particularly when assessing mSAA levels, standardized and verified laboratory techniques were used. To achieve an impartial measurement, all laboratory workers were blinded to participant and outcome data. We reduced information bias by using a standardized data extraction form and having multiple researchers independently validate the data gathered from patient records. We became aware that confounding variables such as maternal age and pre-existing diseases may affect both exposure and outcomes. To identify the influence of mSAA levels on neonatal outcomes, we statistically controlled for these confounding variables using multivariate analysis.

Statistical analysis

Data were analyzed using SPSS version 25 (data management and preliminary analysis; IBM Corp., Armonk, NY) and Python (complex analytical tests and visualization). Continuous variables were expressed as median (interquartile range) and compared between groups using the Mann-Whitney U test. Categorical variables were presented as frequencies (percentages) and compared using Fisher's exact test. The normality of the distribution of continuous variables was assessed using the Shapiro-Wilk test. *P*-value < 0.05 was considered statistically significant. Correlations between continuous variables were assessed using the Pearson or Spearman correlation coefficient, depending on the distribution. In addition, simple linear regression was used to assess the association between mSAA and neonatal leukocyte, CRP, and AS levels. Logistic regression was applied to assess the ORs of specific neonatal outcomes (e.g., AS < 7; RDS) according to levels of SAA.

## Results

Baseline characteristics

Table 1 presents the demographic and clinical characteristics of the participants and their newborns. The median age of the mothers was 30 years, and a history of PTB was reported in 21.2% of the participants. About 21.2% of the participants had medical conditions during pregnancy. The median gestational age at delivery was 31 weeks. The median mSAA level was 6.15 mg/L, and the median AS at birth was 9.

**Table 1:** Baseline characteristics of pregnant women and neonates included in the study. *Continuous variables are reported as median (interquartile range) and categorical variables as number (percentage). IQR, interquartile range

Characteristics	Median (IQR)/Count (percentage)*
Maternal age	30 (7.75)
Preterm birth history	14 (21.2%)
Medical conditions during pregnancy	14 (21.2%)
Gestational age at birth	31 (5.75)
Maternal serum amyloid A (mSAA)	6.15 (11.85)
APGAR score	9 (1)
Neonatal complication	24 (36.4%)
Neonatal leukocytes	15.25 (11.4)
C-reactive protein (CRP) neonates	0.2 (0.45)

Correlation of mSAA with AS in preterm neonates

**Figure 1:**
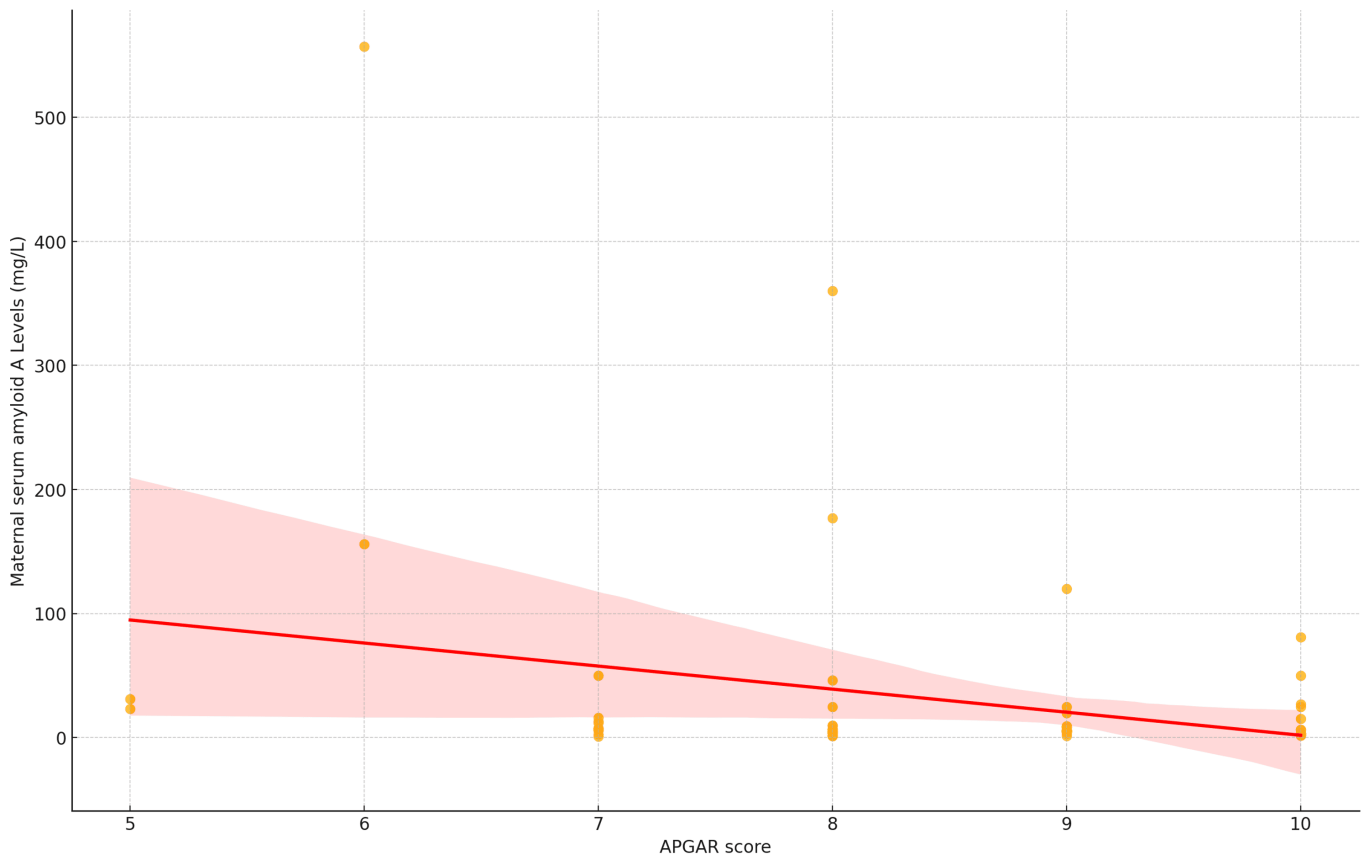
Scatter plot with correlation between APGAR score and maternal serum amyloid A levels (mg/L).

The correlation between the two variables, mSAA and AS, was assessed using the Pearson correlation coefficient. The analysis revealed a statistically significant negative correlation between SAA and APGAR, with a Pearson correlation coefficient of -0.272 (*P* = 0.027). This suggests that as SAA increases, APGAR tends to decrease, although the strength of the correlation is relatively modest, it is still statistically significant (Figure 1).

Furthermore, the linear regression analysis aimed to examine the relationship between the AS and mSAA levels among the newborns included in the study. The model showed a statistically significant negative association between AS and SAA levels, with a regression coefficient of -18.59 (*P *= 0.027). This indicates that, on average, each one-unit increase in AS is associated with an 18.59 mg/L decrease in mSAA levels, suggesting an inverse relationship.

The model intercept was estimated at 187.73 mg/L (*P* = 0.009), representing the predicted SAA level when the AS is zero. The overall model accounted for approximately 7.4% of the variation in SAA levels, as indicated by an R2 of 0.074, with an adjusted R2 of 0.060, indicating a modest effect size. The F-statistic for the model was 5.129 (*P* = 0.0269), supporting the conclusion that the AS is a statistically significant predictor of mSAA levels in this sample (Table 2).

**Table 2:** Summary of linear regression for prediction of APGAR score according to maternal serum amyloid A (mSAA) levels. CI, confidence interval

Predictor	Coefficient	Standard error	*t*-value	*P*-value	95%CI	
					Lower	Upper
Intercept	187.72	69.87	2.68	0.009	48.13	327.32
APGAR score	-18.58	8.20	-2.26	0.02	-34.98	-2.19

**Figure 2:**
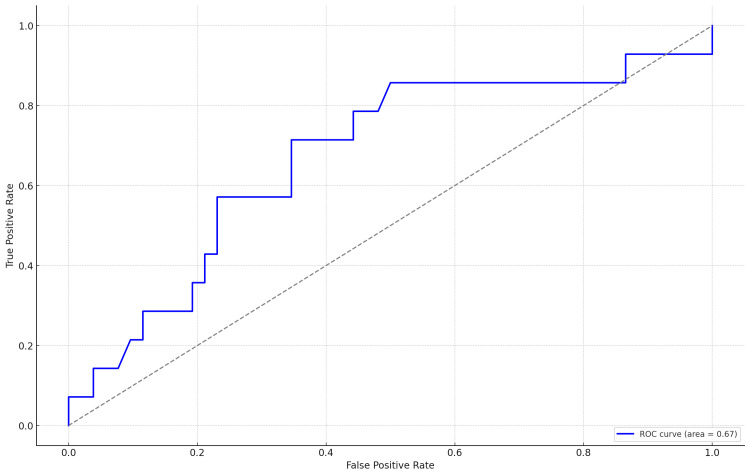
Receiver operating characteristic (ROC) curve for serum amyloid A predicting APGAR score < 7.

The receiver operating characteristic (ROC) analysis, by calculating the area under the curve (AUC), yielded a value of 0.672, indicating a moderate ability of serum amyloid A (SAA) to predict births with low ASs (Figure 2 and Table 3).

**Table 3:** Receiver operating characteristic (ROC) analysis of the predictive value of maternal serum amyloid A for an APGAR score < 7. AUC, area under the curve; SE, standard error; CI, confidence interval

AUC	Standard error	*P*-value	95%CI	
			Lower	Upper
0.672	0.085	0.04	0.505	0.838

Correlation of mSAA with neonatal leukocyte count and CRP

**Figure 3:**
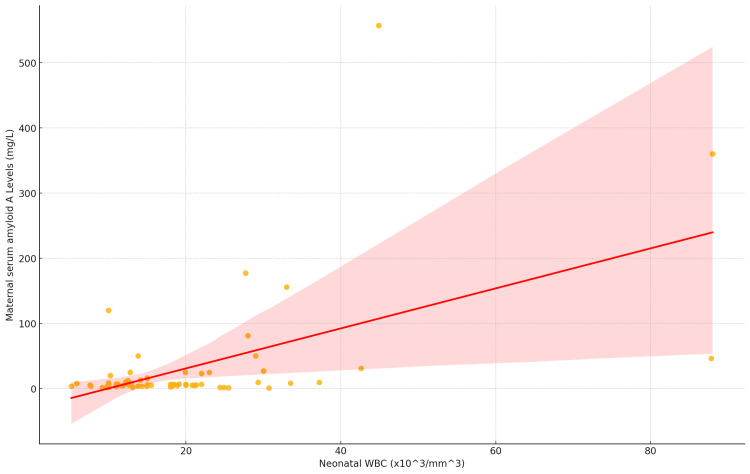
Scatter plot with correlation between neonatal leukocytes score and maternal serum amyloid A levels (mg/L).

The correlation between SAA and leukocytes was assessed using the Pearson correlation coefficient. The analysis revealed a statistically significant positive correlation between SAA and neonatal leukocyte count, with a Pearson correlation coefficient of 0.538 (*P* < 0.001). This indicates a moderate and statistically significant positive relationship, suggesting that higher levels of SAA are associated with higher leukocyte levels (Figure 3).

Linear regression analysis was also performed to examine the relationship between mSAA levels and neonatal leukocyte counts. The model showed a statistically significant positive association, with a regression coefficient of 3.07 (*P *< 0.001), indicating that for each unit increase in leukocyte count (x10^3/mm^3), SAA levels increase by approximately 3.07 mg/L.

The model explained approximately 28.9% of the variability in SAA levels, as reflected by an R2 of 0.289, with an adjusted R2 of 0.278, suggesting a moderate effect size. The F-statistic for the model was 26.02 (*P *< 0.0001), further confirming the statistical significance of leukocyte count as a predictor of SAA levels (Table 4).

**Table 4:** Summary of linear regression for predicting neonatal WBC count based on maternal serum amyloid A (mSAA) levels. WBC, white blood cell; CI, confidence interval

Predictor	Coefficient	Standard error	*t*-value	*P*-value	95% CI	
					Lower	Upper
Intercept	-30.56	14.99	-2.03	0.04	-60.52	-0.61
Neonatal WBC	3.07	0.60	5.10	<0.001	1.86	4.27

**Figure 4:**
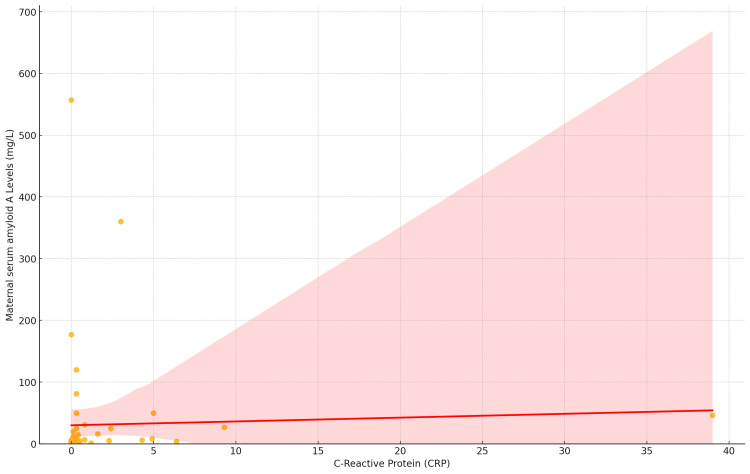
Scatter plot with the correlation between neonatal CRP levels and maternal serum amyloid A levels (mg/L).

The relationship between SAA and CRP was assessed using Spearman's rho correlation coefficient. The results indicate a statistically significant positive correlation between SAA and CRP, with a correlation coefficient of 0.388 (*P* = 0.002). This suggests a moderate positive association between SAA and CRP levels, meaning that higher levels of SAA tend to correspond with higher levels of CRP (Figure 4).

In addition, a linear regression analysis was performed to examine the relationship between mSAA levels and neonatal CRP. The regression coefficient for CRP was 0.62 (*P* = 0.780), indicating that for each unit increase in CRP, SAA levels would increase by an estimated 0.62 mg/L. However, this effect was not statistically significant (Table 5).

**Table 5:** Summary of linear regression for the prediction of neonatal CRP based on maternal serum amyloid A (SAA) levels. CRP, C-reactive protein; CI, confidence interval

Predictor	Coefficient	Standard error	*t*-value	*P*-value	95% CI	
					Lower	Upper
Intercept	30.16	11.99	2.50	0.01	6.00	54.02
Neonatal CRP	0.61	2.20	0.28	0.77	-3.78	5.03

Correlation of mSAA and neonatal respiratory distress

**Figure 5:**
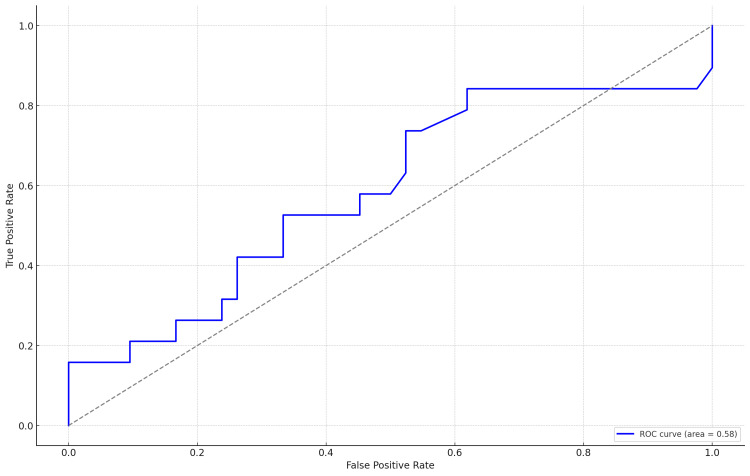
Receiver operating characteristic (ROC) curve for serum amyloid A predicting neonatal respiratory distress syndrome.

ROC analysis assessing mSAA levels as a predictor of neonatal RDS yielded an AUC of 0.58. This value suggests that while there is a statistical association, the predictive accuracy of SAA levels is modest (Figure 5). An AUC closer to 0.5 suggests little to no discriminative ability. Therefore, our findings indicate that SAA alone may not be a strong predictor for RDS.

Furthermore, a binomial multivariate logistic analysis was performed to assess the association between mSAA levels and the risk of neonatal RDS. The model shows an odds ratio (OR) of 1.005, which represents that for each unit (mg/L) increase in mSAA, the risk of neonatal RDS increases by 0.5%, but without statistical significance (Table 6).

**Table 6:** Binomial multivariate logistic regression analysis for the association between maternal SAA and neonatal respiratory distress syndrome. Covariates included in the model were gestational age at birth, APGAR score at 1 minute. SAA, serum amyloid A; *B*, regression coefficient; SE, standard error; OR, odds ratio; CI, confidence interval

Variable	B	SE	OR	*P*-value	95% CI	
					Lower	Upper
SAA	0,005	0.005	1.005	0.31	0.995	1.015

## Discussion

In this cross-sectional study, we investigated the relationship between mSAA levels and various neonatal outcomes, including APGAR score, neonatal leukocyte count, CRP levels, and the occurrence of neonatal RDS.

There was a statistically significant negative correlation between mSAA levels and APGAR score, with a Pearson correlation coefficient of -0.272 (*P* = 0.027). This suggests that higher mSAA levels are associated with lower APGAR scores, indicating that increased maternal inflammation may negatively influence the well-being of the newborn immediately after birth.

A moderate and statistically significant positive correlation was observed between mSAA and neonatal leukocyte counts (*r* = 0.538, *P* < 0.001), indicating that higher mSAA levels are related to increased neonatal leukocyte counts, reflecting a potential inflammatory response in newborns.

The relationship between mSAA levels and neonatal CRP was assessed using the Spearman rho (ρ) coefficient, with a moderate positive correlation (ρ = 0.388, *P* = 0.002). This finding suggests that higher mSAA levels may be associated with increased neonatal CRP levels, indicating a maternal-neonatal inflammatory link.

The significant negative correlation between mSAA and APGAR score suggests that elevated maternal inflammatory markers reflected by SAA may have adverse effects on immediate postpartum neonatal outcomes. In fact, previous studies found that maternal inflammation could have adverse effects on neonatal APGAR scores. The higher levels of inflammatory markers in mothers, such as neutrophil-to-lymphocyte ratio (NLR) and platelet-to-lymphocyte ratio (PLR), are associated with lower APGAR scores [20].

In addition, funisitis and extensive inflammation in the placentas are associated with adverse neonatal outcomes, including an APGAR score of less than 7 at 1 minute, and increased interventional delivery rates [21].

Regarding higher mSAA levels, previous studies have shown an association with neonates admitted to the NICU, but no significant relationship with neonatal death [2]. The referenced study did not detail specific neonatal conditions associated with these NICU admissions and neonatal deaths, highlighting a general predictive value rather than specificity toward particular morbidities.

Regarding neonatal RDS, our study indicated that mSAA levels were not statistically significant predictors of neonatal RDS. Furthermore, Liu et al. showed that a positive maternal inflammatory response (MIR) combined with a positive fetal inflammatory response (FIR) decreases the risk of neonatal RDS [12].

This study has several strengths, including the use of well-defined maternal and neonatal outcomes and the application of robust statistical methods to assess the relationships between maternal inflammation and neonatal health.

Despite its obvious strengths, this study has several limitations. The relatively small sample size (*N* = 66) may be a limiting factor in detecting more subtle associations, particularly in logistic regression analysis for RDS. The cross-sectional nature of the study limits our ability to infer causality. The study was also conducted in a single center, and the small sample size did not allow us to determine the correlation of mSAA with other adverse neonatal outcomes such as mortality or NICU admission. The relatively low AUC prompts a critical evaluation of the role of SAA in clinical practice. Future studies should explore additional biomarkers that could be combined with SAA to develop a more robust predictive model for RDS. Furthermore, one limitation of our current study is the absence of a term delivery control group, which could provide comparative baseline levels of mSAA and enhance the understanding of its predictive value across different pregnancy outcomes.

Longitudinal and multicenter studies are needed to confirm these findings and investigate other possible associations between maternal inflammatory biomarkers and neonatal outcomes to improve prenatal monitoring and early intervention strategies.

## Conclusions

This study shows a significant association between increased mSAA levels and decreased APGAR score, together with an increase in neonatal leukocyte count in preterm infants, suggesting that maternal inflammation may negatively influence the immediate health of the newborn. However, mSAA levels did not demonstrate a significant predictive capacity for neonatal RDS.
